# He Tamariki Kokoti Tau: Families of Indigenous Infants Talk about Their Experiences of Preterm Birth and Neonatal Intensive Care

**DOI:** 10.3390/ijerph18189835

**Published:** 2021-09-18

**Authors:** Anna Adcock, Fiona Cram, Liza Edmonds, Beverley Lawton

**Affiliations:** 1 (Ngāti Mutunga) Te Tātai Hauora o Hine Centre for Women’s Health Research, Te Herenga Waka Victoria University of Wellington, Wellington 6140, New Zealand; 2 (Ngāti Pāhauwera) Katoa Ltd., Auckland 1143, New Zealand; fionac@katoa.net.nz; 3 (Ngāpuhi, Ngāti Whātua) Dunedin Hospital, Southern District Health Board, Dunedin 9016, New Zealand; Liza.Edmonds@southerndhb.govt.nz; 4 Department of Women’s and Children’s Health, University of Otago, Dunedin 9016, New Zealand; 5 (Te Aitanga-a-Hauiti) Te Tātai Hauora o Hine Centre for Women’s Health Research, Te Herenga Waka Victoria University of Wellington, Wellington 6012, New Zealand; bev.lawton@vuw.ac.nz

**Keywords:** cultural competence or cultural safety, family collectives, indigenous health and well-being, Kaupapa Māori Research, NICU, perinatal care, preterm or premature birth, whānau, maternal care, Family Centred Care (FCC), Family Integrated Care (FiCare)

## Abstract

Māori (Indigenous peoples of Aotearoa New Zealand) bear an unequal burden of poor perinatal health outcomes, including preterm birth. An infant arriving preterm disrupts the birth imaginary of whānau (family collectives) and situates them in a foreign health environment that may not be culturally safe and nurturing. A cross-sectional interpretative phenomenological analysis of first interviews with 19 whānau participating in a Kaupapa Māori (by, with, for Māori) qualitative longitudinal study of preterm birth identified themes from their experiences and the meanings they attributed to them. Preterm birth was an emotional roller coaster, with the birth imaginary and anticipated roles disrupted as health practitioners took over the care of their infants. Whānau expressed the desire to be close to their infants, holding them, loving them, nurturing them, and emplacing them within whakapapa (genealogy, continual layering of foundations) networks. When health practitioners or hospital policies inhibited this intimacy by isolating, excluding, or discriminating, whānau were frustrated. Being familiar with hospital routines, staff, peers, infant cares, and being wrapped in wider whānau support were key for whānau coping. Whakawhanaungatanga (processes of establishing relationships) create safe spaces for whānau to be themselves. This quietens the ‘storm’ and returns whānau to a sense of calm, through the reclamation of their environment.

## 1. Introduction

Much attention is paid to the influence that the first five years of life, starting from conception, have on lifelong health and well-being [1]. In Aotearoa (New Zealand), pregnant Māori (Indigenous) women and their infants experience marked inequities in health outcomes [2]. These are part of a wider picture that includes racism, education, financial, and housing inequities for Māori whānau (family collectives) (see Box 1 for a guide of Māori language terms) [3]. Māori whānau experience persistent perinatal inequities, including higher rates of maternal mortality, infant mortality, and preterm birth [4,5].

Preterm (or premature) birth refers to infants born before 37 weeks gestation, and the younger the gestation, the higher the risk of adverse outcomes. Preterm infants are more likely to experience some kind of harm or disability including brain haemorrhage, chronic lung disease, cerebral palsy, and cognitive, visual, or learning impairments [6,7]. Preterm birth is the most common cause of death for infants in Aotearoa and there has been little change in perinatal-related mortality for Māori for at least 11 years [5]. These inequities stem from colonization and the continued colonial project that in Aotearoa breach Te Tiriti o Waitangi (the Treaty of Waitangi), a founding Treaty document [8], and are shared by many Indigenous peoples in similarly high-resource settler-colonial nations [9].

When experiencing preterm birth, Māori are faced with logistical difficulties in many areas of Aotearoa, including an emergency delivery and potentially the transfer of mother and baby to a hospital (with specialist inpatient care) so that baby can be cared for in a neonatal intensive care unit (NICU). In Aotearoa, there are six tertiary level hospitals with NICU facilities. With Māori more likely to live in rural or smaller urban areas without these facilities, Māori face inequitable access to life-saving best practice care [10]. Infants of Māori mothers are more likely to be ‘out-born’, meaning they are born in a facility that does not meet their needs, such as a facility without a NICU, and face increased risk of poor outcomes [11]. Personnel involved in whānau transfers to a tertiary hospital have reported systems seemingly designed for clinical and administrative ease rather than to meet the needs of whānau [10]. Whānau have identified issues related to anxiety, foreign environments, isolation, lack of autonomy, difficulties in accessing government-funded travel assistance, and lack of appropriate information sharing [12,13]. Internationally, similar issues have been reported with non-Indigenous women transferred to tertiary services prior to or following birth [14].

In the hospital, parenting roles are disrupted as health practitioners take over the care of infants, and sometimes also mothers. Power imbalances between health practitioners and whānau are systemically entrenched [15,16], with McBride-Henry and colleagues highlighting how inhospitable hospitals can be for parents of frequently hospitalised young children [16]. Māori whānau in NICUs report isolation, hunger, heightened stress and anxiety for their infants, as they come to terms with the loss of their anticipated birth journey, being parents in the often-alien hospital environment, and juggling external responsibilities, such as work, study, and other children [13,17,18]. Being close to baby and being able to express self-determination in their role as whānau collectives are important [13,18], as are mothers’ desires to nurture their infants through breastfeeding or expressing breast milk [19]. These are enhanced by meaningful communication with staff, especially nurses, and also by wider whānau support and advocacy [18]. While these issues are also reported in neonatal research without an Indigenous focus in Aotearoa [20,21,22,23,24,25] and internationally [26,27,28,29,30,31], Māori whānau also often describe maternal, neonatal, and paediatric care systems that are not culturally responsive [19,32,33]. Research with Indigenous women in other high-income settler-colonial nation states has identified similar birthing issues [34,35,36,37,38].

Box 1 **Guide for the use of te reo Māori (Māori language) terms.**

Aotearoa (New Zealand)

Hapū (kinship group, pregnant)

Iwi (tribal group)

Kāhui Kaumātua (Elders advisory group)

Kai (food)

Kaiāwhina (Māori healthcare support worker/liaison)

Kaitiaki (guardian/advocate)

Karakia (prayers/incantations)

Kaupapa Māori Research (by, with, for Māori research)

Koha (gift)

Māori (Indigenous peoples of Aotearoa New Zealand)

Mātauranga Māori (Māori knowledges)

Papatūānuku (Mother Earth)

Ranginui (Sky Father)

Rōpū Māmā (mothers advisory group)

Taonga tuku iho (gifts that are passed down through generations)

Te ao hurihuri (changing world)

Te ao Māori (Māori world)

Te reo Māori (Māori language)

Te Tiriti o Waitangi (the Treaty of Waitangi)

Ūkaipō (mother, source of sustenance, place/space with a physical/spiritual connection)

Wairuatanga (spirituality)

Whakapapa (genealogy, continual layering of foundations)

Whakawhanaungatanga (processes of establishing relationships)

Whānau (family collective, to give birth)

Whanaungatanga (relationships, connectivity)

Whenua (land, placenta)

For Māori, birthing and reproductive health are central to the continuation of whakapapa (genealogy, continual layering of foundations), and bridge physical and spiritual realms, linking Māori to our primordial parents Papatūānuku (Mother Earth) and Ranginui (Sky Father) [39]. Terms like ‘whānau’ (family collective and to give birth), ‘hapū’ (kinship group and to be pregnant), ‘whenua’ (land and placenta), and ‘ūkaipō’ (night feeding breast—a term for mother; a source of sustenance; and a place/space that a person feels a physical and spiritual connection to) show the importance and interconnectedness of childbirth and connections to place from a Māori worldview [40]. Māori infants and children, as the centre of whānau, embody the aspirations of ancestors past and present, and are literally the future of whānau, hapū, Iwi (tribal groups) and whenua health and well-being [39,41,42]. This vision of intergenerational well-being relies on collective responsibility, a notion that conflicts with Western, neoliberal, and biomedical notions of individual responsibility, gendered parenting roles, and nuclear family structure [41].

Perinatal research studies tend to focus on mothers as the primary care giver [32,43], and previous Kaupapa Māori (by, with, for Māori) studies examining the experience of preterm birth have been retrospective and geographically limited [17,18,19]. Māori researchers have identified that more in-depth research in this area is urgently needed [17]. Responding to these calls, as well as the concerns of a university-based research group’s Kāhui Kaumātua (Elders advisory group), He Tamariki Kokoti Tau—Babies Born Prematurely (a Kaupapa Māori, prospective, qualitative, longitudinal study) was initiated. In this paper, we present an interpretative phenomenological analysis of the first interviews (*n* = 19) conducted with 19 whānau of preterm Māori infants as soon as possible after the birth of their infant/s, exploring their experiences and the meanings they attributed to them.

## 2. Materials and Methods

He Tamariki Kokoti Tau examined the experiences of Māori whānau as they journeyed along preterm care pathways, from birth for one year. In total, 19 whānau (including 19 mothers, 9 fathers, and 13 non-parent whānau) of 21 preterm Māori infants (born 24^+0^–36^+0^ weeks gestation) took part in this study. First interviews took place as soon as possible after birth. Subsequent interviews were conducted at rough milestones, including continued NICU stay, transfer, discharge, settling in at home, and birthday. Sixteen whānau collectives (84%) remained in this study longitudinally, completing 3–6 interviews over approximately 12 months (3 whānau were lost to attrition after the first interview). Over two and a half years, 95 interviews (66 individual, 29 group) were conducted throughout Aotearoa across both North Island and South Island. Ten health providers were also interviewed. This paper takes a cross-sectional look at the first interview with each of the 19 whānau collectives (*n* = 19), following the birth of their preterm infant/s.

### 2.1. Aim

To give voice to the experiences, views and attributions of whānau of preterm Māori infants. The ultimate study goal was to support service transformation to ensure that whānau are supported as they face the joys and challenges of parenting their precious newborn gift.

### 2.2. A Kaupapa Māori (by, with, for Māori) Research Inquiry Paradigm

Kaupapa Māori Research is often described as research that is conducted by Māori, with Māori, for Māori, or, conducted in a Māori way [44]. All four authors are Māori, with the first author (an early career researcher) leading this study and being supported by the co-authors (senior Māori researchers/clinicians).

This Kaupapa Māori Research has a different foundation than Western-oriented research, in that a Māori ontology, or worldview, that is relational—understood through the whakapapa and whanaungatanga (relationships, connectivity) between people and the cosmos—is privileged [45]. So too are the epistemologies, or knowledge construction processes, of mātauranga Māori (Māori knowledges). These support relational axiologies, or ethical values, that are grounded in whakawhanaungatanga (processes of establishing relationships) and wairuatanga (spirituality) [44,46]. Hence, Kaupapa Māori Research methodologies call for practices that are relational, such as, love/respect for people, being seen/known, listening before speaking, collaboration/generosity, being safe/aware/reflective, consultation/feedback, and humility [47]. From this Kaupapa Māori Research inquiry paradigm, the priorities and aspirations of Māori whānau are paramount.

### 2.3. Institutional Ethics

Research involving consumers of healthcare in Aotearoa requires approval from one of the national Health and Disability Ethics Committees (HDECs), and local approvals from participating District Health Boards (DHBs) [48]. Any research that takes place in Aotearoa impacts Māori [49]. For research with Māori, the HDECs expect applicants to explain how the research will benefit Māori, any issues that could potentially arise for Māori, if Kaupapa Māori research methodologies will be used, and what consultation processes (if any) have taken or will take place. Endorsements are also required from locally mandated Māori review groups in participating DHBs. While these institutional ethical review board requirements are important (and were met for this study), ethical research with Indigenous peoples encompasses much more [50], requiring an authentic relationship with ongoing processes of engagement and accountability [51].

### 2.4. Kaupapa Māori Ethics and Governance

As outlined in ‘Te Ara Tika’ (a framework for Māori research ethics), best practice—relationally—involves Māori in governance roles, as kaitiaki (guardians/advocates), fully engaged throughout study design, implementation, and dissemination [49]. Hence, this study was conducted through a Kaupapa Māori Research inquiry paradigm. A Kāhui Kaumātua (a group of knowledge holders of traditional and contemporary Māori cultural practices related to whānau health and well-being) provided the inspiration for this study, and guidance throughout, keeping researchers and participants culturally safe. A Rōpū Māmā (a group of Māori mothers with lived experience of preterm birth) also guided this study to ensure the appropriateness of this study for whānau. By drawing on the expertise of these Kaumātua (Elders) and Māmā (mothers), this study decentred institutional knowledge and research ethics, privileging Māori ways of being, knowing, and relating.

### 2.5. Participant Recruitment

Nineteen mothers (as primary contacts within the hospital setting) of 21 preterm Māori infants (born 24^+0^–36^+6^ weeks gestation) in four large urban tertiary level NICUs were recruited from July 2017 to January 2019, by purposive sampling. They were first approached by a health practitioner (nurse, kaiāwhina (Māori support/liaison), social worker, or registrar) whom they were familiar with in the NICU and were asked for their permission to be contacted about this study. Mothers were contacted by telephone (by A.A.). A brief overview of this study was given, and, if acceptable, a time to meet in person to complete informed consent and potentially be interviewed (by A.A.) was arranged.

### 2.6. Interviews

Focused life story interviews were based around four topic areas: their journey, explanations they had been given, responsiveness of care, and their feelings/coping. This method is compatible with Kaupapa Māori Research as it centres the experiences of whānau and their concerns [47]. Most interviews (*n* = 17) were held in the tertiary hospital setting while the infant/s were still in the NICU (*n* = 1 in a secondary hospital baby unit after transfer back to home region; *n* = 1 at home post-discharge) (median time since birth: 22 days). Whānau could choose to speak in te reo Māori (Māori language). Each meeting began with appropriate rituals of encounter [52], including acknowledging the whānau, sharing of whakapapa, karakia (prayers/incantations) if desired, kai (food), and koha (gift), as well as gaining informed consent.

### 2.7. Whānau Overview

Nineteen whānau collectives (including 19 mothers; 5 fathers; 2 NICU peers; 1 aunt; 1 grandmother) participated in these first interviews. Each whānau was given a pseudonym, named after a bird of Aotearoa. Infant gestational information is outlined below (Table 1). Twelve of the 19 mothers gave birth away from home (90+ minutes’ drive). Age of infants at the time of the first interview, participating adults, and number of siblings are shown in Table 1. Other information is not provided, to protect whānau confidentiality.

### 2.8. Data Analysis

Data were analysed using interpretative phenomenological analysis (IPA), which allows for the bottom-up emergence of themes related to participants’ experiences and the meanings they attribute to them [53]. It has been used previously to explore Māori experiences of health services [13,54]. For this paper, the transcripts from 19 interviews (each with a different whānau following the birth of their preterm infant/s) were annotated with descriptive and later more interpretative notes that gave way to emergent themes. These were then developed and reorganized, through processes such as abstraction and subsumption, until superordinate themes and subordinate themes were finalised [53].

## 3. Results

The results are organized into four superordinate themes, taken from participant quotes, each with three subthemes (Table 2). In the first superordinate theme, Preterm birth is ‘an emotional roller coaster’, we discuss the disruption of preterm birth to the imagined birth journeys of whānau and the emotional toll that this has on whānau as they question themselves and fear for their infant/s, living in uncertainty. The second superordinate theme, In the NICU, ‘I just wanna hold my baby’, explores the strength that whānau draw from their love for their infant/s as taonga tuku iho (gifts that are passed down through generations), and the detrimental impact that separation and denigration of their identities as Māori can have. In the third superordinate theme, In the NICU, ‘it does get quite lonely sometimes’, the idea of separation is further explored, through the experiences of whānau in hospital that leave them feeling isolated and disempowered. The fourth superordinate theme, The importance of familiarity when ‘family is the best support network most of us have’, finds that medical care is relational work that is improved when whānau are connected and confident in themselves and those around them.

### 3.1. Preterm Birth Is ‘An Emotional Roller Coaster’

#### 3.1.1. Temporal Disruption of Preterm Birth

For these whānau, the premature arrival of their infant/s was a temporal disruption to their anticipated birth journeys. Their birth imaginary, a ‘normal’ trajectory of having time to prepare mentally and materially for the birth, was compromised. Whānau described being jolted out of the sense of security that they had in their birth plan. For example, a mother described how she first felt in denial about her impending preterm birth because her previous pregnancy had been ‘normal’—

They called me into hospital and I thought it was just another check-up, so just drove myself to the hospital, turned up and then the doctor at the time said, ‘Oh no, we’re going to keep you in overnight.’ And that’s when they gave me a steroid shot because they said… there could be a chance they’ll have to deliver baby early. So I was only 29 weeks when this all happened. And then at the time I was in denial, because I had a good pregnancy with my first daughter.

(Takahē, mother)

This temporal disruption meant many things for whānau, including not having had time to fully enjoy or come to terms with the pregnancy, and not being eligible for paid parental leave. Some mothers had been monitored during pregnancy, and although they had more time with specialists leading up to delivery, the preterm birth of their infant/s was still shocking and scary. Two mothers who had a history of birth issues and were eligible for specialist care did not receive it during their pregnancy and questioned whether better monitoring could have improved outcomes for their infants.

No whānau felt prepared or ready for the preterm birth. Rather, whānau talked about having too little time with their midwife (visits increase in frequency towards the end of pregnancy); and not being able to wind down from other responsibilities and take time to plan last minute things, such as purchasing car seats and clothing for their infants. This often impacted the whānau collectives’ sense of agency as a process that was expected to be normal and beautiful changed quickly to a time of fear and loss of control. Whānau needed to quickly pivot if they were to adapt to their new birth trajectory. For example, a mother recalled her partner’s calmness in the face of a loss of normalcy—

I went to sleep one night, woke up to go toilet and there was blood everywhere, so my partner rushed me to the hospital, and yeah, baby was born less than an hour later… I freaked out. I was just completely losing it, but my partner was fine, he kept me calm, he kept holding my hand. I nearly broke two of his fingers.

(Tūī, mother)

#### 3.1.2. Self-Doubt and Guilt

For mothers especially, the premature arrival of their infant/s led to a questioning of their own role in how events had played out. When looking back, a sense of guilt or shame was often expressed, as they wondered whether they had done something wrong and were to blame for their infant/s arriving too early. For example, a mother questioned the impact on her body of a previous termination of pregnancy and miscarriages she had experienced before the birth of her infant—

Why did that have to happen to me?… I think my body can only get to a certain point before it rejects, but then I don’t even know.

(Kūaka, mother)

This questioning can be seen as a way of regaining control or agency; the search for an explanation and any indication about how they might have remained on their birth imaginary trajectory. Feelings of guilt were also heightened for mothers who had difficulty producing breast milk, leading to feelings of inadequacy in their ability to be a ‘good mother’ and provide sustenance for their infant/s. They spoke of falling into a depression about this, with it sometimes impacting their relationship with their whānau. One mother described this as the hardest part of her hospital stay—

Getting your breastmilk to come through when baby’s early is really, it’s actually one of the most hardest, stressful things, and I noticed a lot of mums stress about it… I was lying in a bed having the midwives come in and play with my boobs to try and encourage it to come through. Dignity really goes out the window and you’re syringing maybe point five of a ml off.

(Tōrea, mother)

Fathers and other whānau members tried to support the mothers but often felt little control over the situation, perceiving their roles to be less valued by hospital policies and personnel. Fathers also experienced their own challenges if they had other children at home and/or could not take any time off work. For example, a father whose infant was admitted to neonatal intensive care more than 90 minutes’ drive from home described this as being the most difficult thing for him—

I really wanna be here too, while she’s critical, but I feel I can’t be… I’ll be leaving here about one o’clock to catch the kids back at three.

(Pūkeko, father)

#### 3.1.3. Fear for and of Fragile Infants

Feelings of guilt and shame were also compounded when whānau were faced, initially, with their fragile, often technologically dependent, infant/s. When first seeing their infant/s and becoming accustomed to the NICU environment, whānau often described feeling scared to touch, hold, or do ‘infant cares’ (such as changing nappies, checking temperature). For example, a father described his initial fear—

At first I was, like, real, scared to hold him ‘cause he, like, had all these tubes and stuff out of him.

(Tieke, father)

Similarly, a mother related her initial fear to feeling neglectful—

They were so tiny and so fragile. I didn’t want to break them. I thought they were gonna break ‘cause their bones weren’t that strong. So in that area I neglected their duties, ‘cause they were so small, until they got a bit bigger and a bit stronger.

(Kākāriki, mother)

This fear, a lack of confidence in being able to parent their infant/s, created guilt. Such feelings were made worse if they did not feel encouraged by health practitioners to be involved. For example, if health practitioners dictated to them when and who could hold their infant/s or told them off for doing something (supposedly) wrong. It was much appreciated when health practitioners were supportive. For example, a grandmother praised how the NICU staff helped her and her daughter gain confidence with their infant—

I didn’t want to touch him, and I thought that would be really scary [for daughter], and I liked how the nurse encouraged and supported her.

(Toutouwai, grandmother)

The ways in which whānau were communicated with about the needs of their infant/s were important. During early days in the hospital, whānau described feeling overwhelmed by the amount of information they needed to process, while also dealing with the unexpectedness of the situation and a fear for their infant/s. Very quickly they were filled up and needed time for information to settle so they could ask follow-up questions. A mother described this as an emotional rollercoaster—

At times, it’s been like an emotional roller coaster, you know, trying to absorb so many bits of information.

(Kōtuku, mother)

When there was information overload, or a lack of digestible information, mothers and whānau continued to search for explanations about what happened, and potentially continued to look to blame themselves in the midst of this explanation-gap.

### 3.2. In the NICU, ‘I Just Wanna Hold My Baby’

#### 3.2.1. Love as Strength

Whānau expressed love and joy for their infant/s. Some described their pregnancy as a catalyst for positive change in their lives, as a time of encouraging a re-evaluation of priorities and furthering relationship commitments. In some cases, this catalyst may have been heightened by the early birth of their infant/s. For example, where whānau relationships were strengthened as members came together to support the parents, or where parents decided that they wanted to make lifestyle changes, such as quitting smoking in order to support the lung health of their infant/s. Infants were positioned as agentic, with the love for them giving whānau strength and guiding them back onto a birth trajectory that became the new ‘normal’—

I feel safe when I’m with my daughter, yeah, she just brings, she gives me closure. Like it’s such an amazing feeling, you know, something I had always waited for, like yay, I can’t wait to be a mum. And now I’m a mum, I’m like whoah my gosh, like it’s so amazing. I like looking at her, like oh my gosh, I created her, she came from me.

(Kūaka, mother)

#### 3.2.2. Importance of Intimacy

The love that whānau felt for their infant/s meant that they wanted to be close to them to provide care. Being close helped them to learn about the hospital routine and become accustomed to looking after their infant/s. Being located close, where they could easily see their infant/s, have skin to skin cuddles, and express milk or breastfeed were some of the key ways that whānau connected with their infant/s after recovering from initial fears and trauma. For example, a mother described the emotional toll of not being able to hold her infant for days—

It was like nearly a week before I got to hold her the first time. So that was just killing me, everyday coming in, and like, I just wanna hold my baby.

(Kōmako, mother)

Another mother, who was not given hospital accommodation, spoke about the pain of having to leave her infant when she went home—

So they tell me he’s doing good and then they tell me he’s not doing good. Um, it’s hard. It’s tiring. I just wanna be there for my son, 24/7, like I really just wanna go in there and sit with him.

(Kiwi, mother)

Being located close to their infant/s was seen as important for successful expressing and bonding, with the two intertwined—

Yeah [my breasts are] good, cause I milk them a whole lot, and babies feeding now, and I have a room here now so I can stay, I can stay with baby and do night feeds.

(Pītoitoi, mother)

Being able to hold their infant/s was seen as a privilege by whānau, especially when it was dictated by health practitioners and not always available to non-parent whānau members. Some health practitioners or units allowed this while others prohibited grandparents or other whānau members from holding infant/s and/or visiting the NICU alone. This was frustrating for those whānau who wanted that extra support. For a young first-time mother who lived at home with her mother and siblings (Toutouwai whānau), not being able to introduce her youngest sibling to her infant was seen as culturally unsafe care. In one conversation, three generations of the whānau were noted as being upset by this—

Mother: Yeah and then we went down and took my younger sister with us but they told her that she wasn’t allowed to see him because she’s eight years old so my little sister came all the way down and wasn’t even allowed to see him.

Grandmother: And that was one of our concerns, they said it can only be a direct sibling.

Mother: Yeah, only direct siblings are allowed to see the babies.

Grandmother: To me that’s the White way of thinking, we’re Māori.

Mother: My nanny was very mad.

#### 3.2.3. Celebrating Whakapapa and Wairuatanga

As well as holding, feeding, and caring for the needs of their infant/s, whānau connected with their infant/s through whakapapa and spiritual practices. It was common for whānau to talk about their practices for connecting their infant/s within their whakapapa. They related their infant/s to their ancestors (past and present) through naming and associating, a process of intergenerational whakawhanaungatanga. Giving infants whakapapa names was seen as an important way of linking them to their ancestors. Some whānau shared concerns about these important names being disrespected by health practitioners, who would mispronounce or avoid saying names rather than attempting to learn them. For example, a mother and father (Tākapu whānau) talked about not wanting to tell the name of their infant to health practitioners—

Mother: They’ve been saying that our baby is the baby with no name because we haven’t named him yet. We have named him but we don’t want to share that straight away. It’s important to us, it’s special to us.

Father: You know, it’s also that fear that they’d start making fun of our kid.

Mother: It’s gonna be a Māori name because already two nurses were having a joke about my name not knowing whether they’re pronouncing it right or not.

Some whānau, who experienced health practitioners making an effort to get names right, talked about being impressed and feeling respected. As well as naming practices, whānau also shared other practices that relate to the spiritual, or wairuatanga, such as karakia (incantations). While some whānau had members who were able to say karakia or prayers for their infant/s (and mother) in the hospital, other whānau appreciated the offer of hospital-based Māori spiritual services. For one whānau who lost an infant, a Māori Chaplain was able to support—

They asked me if I wanted a priest and I was like, but I want a Māori one… They came in and they did a karakia for him as his farewell journey.

(Kākāriki, mother)

Other spiritual practices that cement the place of infant/s within the universe include whenua ki te whenua (returning the placenta to the earth) and returning the belly button and other body parts to the earth as well. Most whānau emphasised the importance of these practices for themselves and their infant/s. Concerns about preterm birth interrupting these practices included fear of the placenta being sent for testing and not being returned to the whānau before transfer to another hospital/home (due to previous experiences of this), and it being lost or accidentally destroyed. For example, one whānau recounted their grief upon discovering that their placenta had been destroyed—

They couldn’t find it, they couldn’t find it. We got a call about it not so long ago and then went up to get it and they said that they don’t get them if they’ve been researched cause they sent it away… I think it was just last week my cousin got a phone call telling us to pick it up before twelve days and they told me they didn’t know where they sent it, so I pretty much gave up.

(Kea, mother)

Whānau also worried about what chemicals the placenta had been treated with when sent for testing (because it is usually buried with a tree or other plant), and not being able to bury their placenta at their ancestral homeland (because of physical distance or disconnection). As the centre of whanau, infants embody the hopes and aspirations of their ancestors both past and present, and represent taonga tuku iho—gifts that are passed down through generations. Infants are crucial to the continuance of whakapapa, and, therefore, the vitality of whānau, hapū, and Iwi. As such, practices that hold space for infants—physical intimacy, nurturing, spiritual safety, and connecting with place—were ways that these whānau enacted agency as whānau.

### 3.3. In the NICU, ‘It Does Get Quite Lonely Sometimes’

#### 3.3.1. Isolation

Whānau talked about the detrimental impact of birthing a preterm infant, spending much longer than anticipated in the hospital and dealing with ongoing health concerns. While, in most cases provisions would be made so that the mother could stay with their infant/s in the hospital, or in accommodation close by, mothers often spoke of being isolated from their support networks. For example, a mother described the emotional toll that prolonged time alone in the NICU had on her—

I just feel like it’s been real rough emotionally the last couple of weeks… Well, I just try and keep myself occupied, you know, just by doing her, which is fine, but it does get quite lonely sometimes.

(Toroa, mother)

Fathers, while usually being considered primary caregivers and thus given full access to the NICU, often had other employment and/or childcare responsibilities. In most cases, anyone who was not a parent was not allowed to visit the NICU outside of strict visiting hours and visiting rules. Rules around at what age children could visit, and when, impacted whānau who had other children (not always biological siblings) to consider. For example, a mother who was breastfeeding a toddler talked about added pressure and guilt associated with feeling like she was abandoning him (with her partner) while she spent all day in the NICU and her elder children were sent to stay with her parents—

[My partner] looks after our son, cause he’s not allowed in here at all and we had no other way to take care of him. Like they were saying, ‘Ah just get one of your family members to come and look after him,’ but it’s not that easy.

(Kea, mother)

This was particularly hard for whānau when the mother was transferred to or birthed in another region, away from their home place, or where non-parent whānau members were key support people, for example, if the father was away or not involved. This created stress for whānau and further feelings of guilt about having to choose where to be and what trade-offs to make in order to support the infant/s and parents. Transfers to other regions in most (all but one) cases also meant that mothers were transferred alone, and their support people had to find their own transport to the hospital later. That was usually described as a terrifying experience by the mothers, for example—

No, No, just myself, you were only allowed the patient in the ambulance… But I know that [my mother] was upset because she was a bit lost at what to do and she wanted to come as well but. Yes, it was a bit freaky being on my own but there weren’t really any other options.

(Kōtuku, mother)

#### 3.3.2. Inhospitable Spaces

Such stress was compounded by environments, within the hospital and accommodation facilities, that whānau found to be inhospitable or racist. Some whānau described going without food, not being provided clean/safe accommodation, and transport issues such as receiving large parking tickets outside the hospital. Experiences of receiving culturally unsafe care, being treated suspiciously like they were thieves, or having assumptions made about them created discomfort. Whānau described having assumptions made about their smoking status, their ethnicity, and about their parenting skills or relationships. Some also talked about being treated poorly because they were young and/or Māori. For example, a mother talked about feeling dehumanised by her treatment at one hospital—

Because they don’t treat you like you’re a person you’re just a paycheck, like they’re rude, you know, they just make you feel uncomfortable.

(Pīhoihoi, mother)

Not understanding the importance of whānau collectives, and alienating infants and their mothers from their wider support structures due to hospital policies or health practitioner practices further isolated whānau. For a whānau who were being supported in the NICU by the aunt of their infant, the insinuation that the aunt should not be there and the father was not doing enough resulted in them making a complaint to management—

And so one of the nurses waited for me and my sister to leave and then she cornered my partner and were saying, ‘Oh do you feel comfortable with her being here? ‘Cause if you don’t feel comfortable, you just let one of us know and we can get her to leave.’ And my partner was like, ‘Well, what are you talking about? She’s my sister and she’s helping’.

(Pītoitoi, mother)

As well as experiencing microaggressions, receiving mixed messages about hospital expectations and instructions for caring for their infant/s sometimes resulted in situations where whānau were admonished for things unfairly and it left them feeling frustrated—

Yeah, it happens all the time in here. Yeah, it’s a frustrating thing being told one thing and then, you know, you go do it and then another nurse is like, “No, it’s got to be this way,” you know, it’s like, “Well we just got told that way.” And they’re like, “No you’ve got to do it like that.” And so, it’s really frustrating.

(Kūaka, mother)

#### 3.3.3. Lack of Autonomy

Not quite knowing where they stood with health practitioners or certain hospital routines/schedules related to what whānau often described as a lack of autonomy, and the taking over of care by health practitioners—feeling like their infants were not really theirs. They described living on hospital time, frequently having to wait for more information and for health practitioners, and sometimes having the expected routine changed and so missing things that were important to them. Not feeling like they were able to determine their routine within the NICU was posed as encouraging disengagement. For example, a mother talked about a feeling of uselessness—

There’s lots of babies but their mums aren’t here. Cause sometimes you feel like you’re not really doing anything, cause you just sit on the chair.

(Kererū, mother)

A lack of autonomy, in having the care of their infant/s taken over by health practitioners, was further exacerbated when whānau were dictated to about when they could hold their infant/s, and what they could do (as discussed earlier), and in more extreme examples, when whānau were given incorrect information, were prevented from being with their infant/s, or were informed that their infant/s would be taken out of their care. While these examples were few, their effects were severe. They highlight the power that is in the hands of health practitioners to cause unnecessary added trauma to whānau who are already experiencing potentially one of the most stressful moments of their life.

### 3.4. The Importance of Familiarity When ‘Family Is the Best Support Network Most of Us Have’

#### 3.4.1. Importance of Whānau and Peer Support

The importance of extended whānau support was emphasised in all interviews. Non-parent whānau members often made great efforts to support the newborn infant/s and their mother and father. They ran errands, picked up and dropped off parents, delivered food and clothing, and advocated for parents to receive entitlements in the hospital, such as meal vouchers, accommodation, or transport assistance. Importantly, they were often the emotional support for parents, and especially mothers, during trying times. Not only did they show up in person often if they could, they would be there on phone calls and video calls, checking in with parents frequently. For example, a mother talked about the benefits, to her spirit, of being able to have one of her two elder children with her—

No I haven’t been alone since I’ve been here. It’s gonna come, I know it’s gonna come, because my kids will go away on holiday with their grandparents. But yeah, no I haven’t been alone yet. I’ve had one of my children with me, and my partner comes up every weekend, so, yeah, that’ll be interesting.

(Kōkako, mother)

For this whānau, who had been transferred to another region, having that support there for the mother made a big difference. However, in other cases, a lack of whānau support due to hospital policy, distance, or whānau challenges, was mourned. Overall, there was variation in the ways in which non-parent whānau were able to support parents and infants, both across and within hospitals. Sometimes non-parent whānau members were able to take on key support roles, spending time with the infant/s, allowing the parents to rest. In other instances, these whānau members were restricted (as discussed earlier), or they were unable to be there physically because of a lack of support available, especially if they lived a great distance away. For example, a mother who had been transferred from a rural region described the challenges of being far from her support networks—

I think family is the best support network most of us have and I’d love to have more of my family here.

(Tōrea, mother)

For times when extended whānau support was less available, peer support was identified as important for parents coping with their infant/s being in intensive care. Other parents who were experiencing similar circumstances were seen as being some of the few people who could really understand what was going on for parents (even more so than whānau). Running into peers in the family room while making a hot drink, seeing them around in the hospital-arranged accommodation, or even meeting them on hospital bus services to and from the NICU, were key ways that parents became familiar with the hospital environment. A mother talked about how, as having spent weeks already around the hospital for their infant, she and her partner would try to engage with newer parents to let them know key bits of information, and encourage them to ask questions and engage with nursing staff—

We introduce ourselves, just let them know that we’ve all been where they’ve been sitting, like the new parents. Until they decide basically what’s going on with their baby, we just tell them, just talk to the nurses.

(Pūkeko, mother)

While another mother talked about how she had been supported to feel welcome mostly by her peers rather than hospital staff—

We found out about [travel assistance] through other parents… and we went to reception and started asking questions… it was more the parents that made me feel welcome, not so much the nurses and the doctors, it was the parents.

(Kākāriki, mother)

#### 3.4.2. Culturally Safe Care

Another key avenue of support for parents and whānau was culturally safe care. When health practitioners were loving and empathetic, caring for parents and whānau as well as their infant/s, whānau felt comfortable and confident to participate in hospital routines. Continuity of care was deemed by whānau as good for developing relationships with health practitioners on a social/relational level. Whānau talked about preferences for less changeovers of staff—

Nah I feel like they’re really good people here, really nice and caring. I think the only thing, that if I was to change something, I would probably, instead of switching up the nurses so many times, like maybe the same nurses could stay at that same time. Cause there’s just too many swaps.

(Pītoitoi, aunt)

Continuity of care was also seen as positive for the health and well-being of their infant/s, who were said to gain from health practitioners knowing them and their cues. This would enable issues to be picked up on earlier and whānau to feel more comfortable leaving their infant/s in their care. However, when issues were had with certain health practitioners, whānau appreciated being able to request that they were not involved in the care of their infant/s. Health practitioners who encouraged whānau to take part in, and especially lead, the care of their infant/s in a supportive way were appreciated as champions of care. For example, a young couple (Tākapu whānau) talked about how some of the staff in the NICU, where they had been transferred for a period, treated them like human beings—

Father: There’s definitely some good ones there.

Mother: They’d like to joke around with you and make sure you were alright.

Father: *They made Mum quite happy*.

Mother: They don’t want to pressure us at all.

Father: You know, the way they treat us, it’s just like human beings.

Other whānau talked about feeling the love of certain health practitioners, and knowing when they really cared for both infant/s and wider whānau. These key support people enable whānau to feel confident in the medical care of their infant/s, and to feel comfortable in the often-alien environment of neonatal intensive care. They became like family for whānau, with many whānau talking about those practitioners in familial terms—as people that they would miss and want to visit again.

#### 3.4.3. Becoming Expert

As whānau became more confident in the hospital setting, learning routines and getting used to the needs of their preterm infant/s, they often moved from feeling anxious to feeling familiar and normal in the abnormal setting that is the NICU—

They’re really good with us, they just, it literally is like being at home now aye.

(Pīwaiwaka, mother)

As they spoke of their infant/s and the infant cares that they had become more involved with, their discussion indicated how they had become expert in the medical needs of their infant/s, often describing in great detail, the procedures that their infant/s had and the technology involved. For example, a mother (Pīhoihoi whānau) talked with another mother (a NICU peer) about the change from being scared of all the tubes and wires to becoming an expert of their infant’s cares—

NICU peer: ‘Cause, yeah man, we’re experts. We are now.

Mother: It’s like the first thing you’ve got to learn when you actually have your child.

This knowledge base was supported by the culturally competent/safe care of health practitioners who took the time to talk through things with whānau, such as when they talked to whānau on an understandable, even playful level—joking around with them and putting them at ease. When whānau felt well supported, whether by extended whānau, health practitioners, or peers (or preferably all), the hospital environment became more like home. Becoming expert, feeling familiar, and being valued were important for coping with times that were difficult. As the hospital setting became more normalised, whānau positioned themselves as agentic, and as being resilient.

## 4. Discussion

This is the beginning of an ‘in-time’ longitudinal study and thus the beginning of a long-term relationship with whānau, where they shared generously about their experiences and feelings. Such generosity was most likely because, being a Kaupapa Māori Research study, it was based in te ao Māori (a Māori world), looking out with them through a Māori lens into te ao hurihuri (their changing world). It is the first Kaupapa Māori study, and, to the best of our knowledge, the first ‘by, with, and for’ Indigenous study, to do so. 

The findings from this analysis show the importance of whānau of preterm Māori infants receiving culturally safe care to enable them to acclimatize to their new ‘normal’ trajectory—as Māori, as whānau collectives. A birth imaginary for Māori is sourced within taonga tuku iho, knowledge passed down through generations that nurtures and prepares expectant mothers and whānau so they are well placed to welcome their precious newborn. This knowledge from the past—mātauranga Māori—exists in the present and contributes to the future because it enables us to understand who we were, who we are, and who we may become [55]. When the birth imaginary is disrupted by preterm birth, and whānau find themselves reliant on a health care system not known for its cultural responsiveness, it is as if whānau who were prepared and packed for a te ao Māori journey were then diverted into a te ao hurihuri encounter that no amount of travel guide reading could have prepared them for.

As reported in previous research, for these whānau, this new trajectory, te ao hurihuri, was an emotional roller coaster, as they encountered new medical terminology, foreign environments, and uncertainties [17,18,19]. At the same time, accessing non-medical support, such as transport and even meals was often overly complicated and frustrating [12,56]. Whānau were left feeling anxious and unprepared. This stress was then compounded if whānau were not welcomed and reassured with culturally safe care, but were instead left to struggle against a Eurocentric system that has its own ways and habits that make whānau feel excluded and more vulnerable. Whānau have so much to deal with when they have a preterm infant, with potential cumulative effects of care that is not culturally safe adding to the trauma. For those whānau transferred away from their home especially, the financial burden of preterm birth can have devastating consequences for the whole whānau. Travel expenses, unpaid time off work, and variable access to accommodation and support geographically all contributed to stress. However, whānau spoke of their infant/s giving them the strength to face their new normal, and that they became experts of their infants within this environment is noteworthy.

By speaking with multiple whānau members, we heard how the phenomenon of preterm birth impacts whānau roles differently. Mothers often expressed guilt and feeling as though they were failing in their maternal role. Their bodies and minds had been thrown into this whirlwind, with both needing time to come to terms with what had happened. When fathers or wider whānau members were excluded from participation in the care of their infant/s, this increased the pressure on mothers to be ‘perfect’ and always present, which is not realistic. Fathers felt left out, taking longer to adjust to parenting and feel comfortable getting involved in infant cares. Being separated from whānau support networks created a perfect storm for mothers and fathers to experience emotional distress. For their wider whānau members, disconnection was made even worse with hospital policies that excluded them, and they often felt torn. Parents and their whānau do not wish to be perceived as isolated individuals, but rather as situated within complex social networks. Health services taking the time to build relationships with these wider whānau networks is, for Māori, “a cultural imperative” (p. 2) [57], and is completely necessary, as they are the ones who will take their infant/s home.

Birth ceremonies and practices helped whānau to create and hold space for their infants in a way that secures them in their cultural identity. Giving whakapapa names, saying karakia, and burying the placenta helped to connect infants to their expansive whakapapa—that spans right to Papatūānuku and Ranginui—keeping them spiritually and physically safe. When these practices were not respected or were inhibited, whānau expressed great sadness.

The literature strongly suggests that some of the experiences these participants had of preterm birth are shared by non-Indigenous women and families, for example, mothers’ guilt about not being able to feed their infants in the NICU [31]. What we are adding here is the intensification of these things, like self-doubt and guilt, by virtue of these women and whānau being Māori, living in a stigmatizing society. The impact that a lack of culturally safe care and the lack of cultural understanding of what a baby is for Māori as whānau collectives heightens such negative experiences [19]. Yet, the whānau were so generous to acknowledge and praise the positive things that they did experience.

Health practitioners who provided empathetic, culturally safe care helped parents and whānau feel at home. They became like family, the process of which—whakawhanaungatanga—creates a safe space for whānau to be themselves. This is what quietens the ‘storm’ and returns whānau to a sense of calm, through the reclamation of their environment. This can be likened to the concept of Birthing on Country, a culturally safe model of maternity care in Australia, which shows great potential for enacting these principles to translate into health gains for Indigenous families, including reducing rates of preterm birth [36,38]. In this study, health practitioners who created space for Māori to be as Māori when experiencing preterm birth—by respecting whānau knowledge, involving, encouraging, and being welcoming—helped whānau conserve their energy for what was important to them, i.e., being there for their precious newborn gift. With this familiarisation and comfort, feeling at home, came a tipping of the scales in terms of power dynamics, where whānau felt autonomous.

The involvement of whānau as expert knowledge holders and as important caregivers has been shown to improve the health outcomes of preterm infants, and recognised in neonatal care models such as Family Centred Care (FCC) and Family Integrated Care (FICare) [25,58,59]. Moving away from the dichotomy of traditional, medicalized NICU parent-infant dyads to a more holistic view of whānau could include things such as reviewing visiting policies and the structural environment, e.g., being able to stay near and with their infant/s along with extended whānau. In theory, FCC and FiCARE are supposed to enhance autonomy and whānau involvement, but how this translates from the traditional hierarchical medical approach requires systemic changes to care that are long overdue [60].

The feelings of loss and the roller coaster of a NICU admission must be acknowledged. We recommend the strengthening of maternal and infant mental health supports in the NICU as routine care, such as clinical psychologists available for both whānau and health practitioners. This would be an opportunity to strengthen resilience for whānau and educate staff around how to do this in an appropriate way without stigmatizing mental health [61,62]. Secure attachment predicts long-term better outcomes [61,62], and NICU teams have opportunities to support this, through interventions like skin-to-skin care (also called kangaroo care), of which there is clear evidence of benefits [63,64]. However, how often and for how long whānau are able to do this is variable. NICUs need to not only allow, but encourage and promote skin-to-skin care [22].

Ultimately, culturally safe care is best evidence care [38,57,65,66]. Challenging the NICU to ensure this is a fundamental right and upskilling staff to ensure this occurs always, *with all care*, should be mandated, such as from the national Newborn Clinical Network (clinical leadership for newborn services). Simple things, like names and respect, cost nothing but mean so much. Recognizing the journeys that whānau take to become an expert of their infant/s, and valuing their expertise in the NICU would surely help with all of the above. Models like FiCARE can help, but this has been introduced without an Indigenous lens, and implemented variably across the country without a focus on cultural responsiveness [59]. Perhaps in Aotearoa we need to move beyond FICare (and models that do not centre Māori aspirations) and towards WhICare—that is, Whānau Integrated Care. WhICare would take into account the unique and sovereign position of Māori as Indigenous peoples, as tribal peoples, and restore the power balance that was guaranteed in Te Tiriti o Waitangi.

Limitations of this study include that given how hospital settings are organized and the outside responsibilities of other whānau members, most of the participants were mothers (especially at these first interviews). More research could explore the views and experiences of wider whānau members, including siblings of preterm Māori infants.

Key strengths include its Indigenous—Kaupapa Māori Research—lens; that it was prospective and longitudinal—walking alongside whānau on their preterm care pathways, exploring their lived realities in-depth—which had not been done before; and that it sought the views of diverse whānau from across multiple regions of Aotearoa. These findings undoubtedly speak to the lived experiences of many whānau finding themselves in similar, unfamiliar health care surroundings, who need to surrender care of a loved one to health practitioners who operate within a culture that is foreign territory for Māori. These can be environments with few and possibly no ‘sign posts’ of culturally safe care by culturally competent health practitioners, or, champions. What we did hear about champions, though, indicates there is potential for culturally safe care to be scaled up across health care systems so that Māori whānau and other under-served groups find people who are on their side. These champions, who can support whānau and systemic transformation (over time), are the way forward.

## 5. Conclusions

This research sheds light on the lived realities of whānau as they journey along preterm care pathways. We recommend health services listen to these whānau and consider how care provision can be optimized with whānau. In particular, the potential of pregnancy and birth as a catalyst for positive change, and the special position of Māori infants as taonga tuku iho need to be acknowledged. Whānau are experts in their own worlds and of their infants, and ought to be listened to and supported to find the intimacy with their infants that gives them strength. Whakapapa and wairuatanga practices that emplace preterm Māori infants firmly within intergenerational relationships must be celebrated and protected. For whānau to feel at home, for the chaos to quieten, they should be wrapped up in love, by wider whānau, peers, and health practitioner champions. These strengths, as identified by whānau, contribute to whānau well-being and should be scaled up to inform Māori-centred models of neonatal care. This should be of particular interest to maternity and neonatal services and policy makers, as well as other providers who support whānau in Aotearoa. The results may also be of interest to stakeholders in other high-income settler-colonial nation states where Indigenous families are disproportionately impacted by preterm birth and inequities in perinatal health outcomes.

## Figures and Tables

**Table 1:** Whānau information.

Category of Preterm	Whānau Pseudonym	Infant/s	Infant/s Age at First Interview	Adult Participants in the First Interview	Siblings
Extremely preterm (<28^+0^ weeks gestation)	Kōmako	1	3 weeks	Mother	0
	Kiwi	1 *	3 weeks	Mother, father	4
	Kererū	2	11 days	Mother	0
	Kea	1 *	6 weeks	Mother	2
	Kōkako	1 *	2 weeks	Mother	2
	Kākāriki	2 *	15 months	Mother	1
	Kōtuku	1 *	1 month	Mother	0
	Kūaka	1	4 months	Mother	0
Very preterm (28^+0^–31^+6^ weeks)	Tūī	1	7 weeks	Mother	3
	Tieke	1	6 weeks	Mother, father	0
	Tākapu	1 *	3 weeks	Mother, father	0
	Toroa	1 *	3 weeks	Mother	0
	Takahē	1 *	2 weeks	Mother	1
	Toutouwai	1 *	3 weeks	Mother, grandmother	0
	Tōrea	1 *	3 weeks	Mother	5
Moderately preterm (32^+0^–36^+6^ weeks)	Pūkeko	1 *	6 weeks	Mother, father	5
	Pīhoihoi	1 *	1 month	Mother, NICU peer (mother), NICU peer (father)	4
	Pīwaiwaka	1	10 days	Mother, father	0
	Pītoitoi	1	1 week	Mother, aunt	0

* = Born away from home (from a rural or different region).

**Table 2:** Superordinate themes and subordinate themes.

Superordinate Themes	Subordinate Themes
3.1 Preterm Birth Is ‘An Emotional Roller Coaster’	3.1.1 Temporal Disruption of Preterm Birth 3.1.2 Self-Doubt and Guilt 3.1.3 Fear for and of Fragile Infants
3.2 In the NICU, ‘I Just Wanna Hold My Baby’	3.2.1 Love as Strength 3.2.2 Importance of Intimacy 3.2.3 Celebrating Whakapapa and Wairuatanga
3.3 In the NICU, ‘It Does Get Quite Lonely Sometimes’	3.3.1 Isolation 3.3.2 Inhospitable Spaces 3.3.3 Lack of Autonomy
3.4 The Importance of Familiarity When ‘Family Is the Best Support Network Most of Us Have’	3.4.1 Importance of Whānau and Peer Support 3.4.2 Culturally Safe Care 3.4.3 Becoming Expert

## Data Availability

Data cannot be made publicly available for ethical reasons—participants did not consent to their data being made publicly available, and the publication of full interview transcripts would compromise confidentiality.
